# CRISPR-Cas12a-Based Isothermal Detection of *Mammarenavirus machupoense* Virus: Optimization and Evaluation of Multiplex Capability

**DOI:** 10.3390/ijms26199754

**Published:** 2025-10-07

**Authors:** Marina A. Kapitonova, Anna V. Shabalina, Vladimir G. Dedkov, Anna S. Dolgova

**Affiliations:** 1 Laboratory of Pathogen Molecular Genetics, St. Petersburg Pasteur Institute, St. Petersburg 197101, Russia; kapitonova@pasteurorg.ru (M.A.K.); shabalina@pasteurorg.ru (A.V.S.); vgdedkov@yandex.ru (V.G.D.); 2 Martsinovsky Institute of Medical Parasitology, Tropical and Vector Borne Diseases, First Moscow State Medical University (Sechenov University), Moscow 119048, Russia

**Keywords:** *Mammarenavirus machupoense*, Bolivian hemorrhagic fever, CRISPR-Cas12a, DETECTR, RPA, isothermal amplification

## Abstract

Bolivian hemorrhagic fever (BHF) is a zoonotic disease caused by *Mammarenavirus machupoense* (MACV) featuring severe neurological and hemorrhagic symptoms and a high mortality rate. BHF is usually diagnosed by serological tests or real-time polymerase chain reaction (RT-PCR); these methods are often inaccessible in endemic regions due to a lack of laboratory infrastructure, creating a demand for sensitive and rapid equipment-free alternatives. Here, we present an isothermal method for MACV nucleic acid detection based on the Cas12a-based DETECTR system combined with recombinase polymerase amplification (RPA) in a single tube: the RT-RPA/DETECTR assay. We demonstrate the possibility of using more than one primer set for the simultaneous detection of MACV genetic variants containing multiple point mutations. The method was optimized and tested using specially developed virus-like armored particles containing the target sequence. The multiplex RT-RPA/DETECTR method achieved a limit of detection of approximately 5 × 10^4^ copies/ mL (80 aM) of armored particles. The method was validated using clinical samples spiked with virus-like particles. The assay proved to be selective and reliable in detecting certain nucleotide substitutions simultaneously.

## 1. Introduction

*Mammarenavirus machupoense* (MACV) is the etiologic agent of Bolivian hemorrhagic fever (BHF) [1,2]. MACV is a negative-sense RNA virus belonging to the *Arenaviridae* family. The natural reservoir of *M. machupoense* is the large vesper mouse, *Calomys callosus* (Rengger, 1830) (family *Cricetidae)*, found in countries of South America. Since the first outbreak in 1959–1964, the virus has been detected in Bolivia in the provinces of Mamoré, Iténez, and Yacuma of the Beni Department and in the Province of Cercado of the Cochabamba Department. Transmission of MACV from *C. callosus* to humans has been identified as occurring through: aerosols from excrements or secretions of infected animals; consumption of contaminated food; or direct contact with virus-contaminated material. There have also been confirmed cases of nosocomial, human-to-human viral transmission between patient family members [1].

The BHF incubation period is about 3–16 days. The prodromal period lasts 1–5 days. After this, within a week after the prodromal period, about a third of patients develop severe neurological and hemorrhagic symptoms [1]. BHF mortality during outbreaks has been estimated to be 25–33%.

Since 2005, the number of BHF cases has increased. There have been 331 cases with 22 deaths in the 2005–2013 time period [1,3,4]. This may reflect improvements in diagnosis. It might also be associated with an increase in the actual number of infected people, potentially as a consequence of population growth or expansion of agricultural areas. Like other hemorrhagic fever viral pathogens, MACV is currently classified as Biosafety Level-4 (BSL-4) [1,2].

Generally, laboratory diagnostics of BHF are possible after the third day of infection, corresponding with the development of viremia and specific antibodies. However, there are no perfect methods for MACV detection, and all of them have some disadvantages or natural limits. For example, viral isolation and neutralization antibody tests require BSL-4 conditions. Serological tests, such as enzyme immunoassays (ELISA) for IgM and IgG antibodies against MACV, can exhibit cross-reactivity with other arenaviruses. RT-PCR (reverse transcription polymerase chain reaction) is a commonly used tool for viral genetic material detection, which allows distinguishing MACV from other viral hemorrhagic fevers. Despite all the widely known advantages of RT-PCR tests, they are still limited to specialized laboratories with thermocycling devices. These are lacking in endemic Bolivian regions. Here, we report the development and initial testing of an isothermal MACV detection assay based on the DETECTR system with Cas12a nuclease and recombinase polymerase amplification with reverse transcription (RT-RPA).

**Figure 1:**
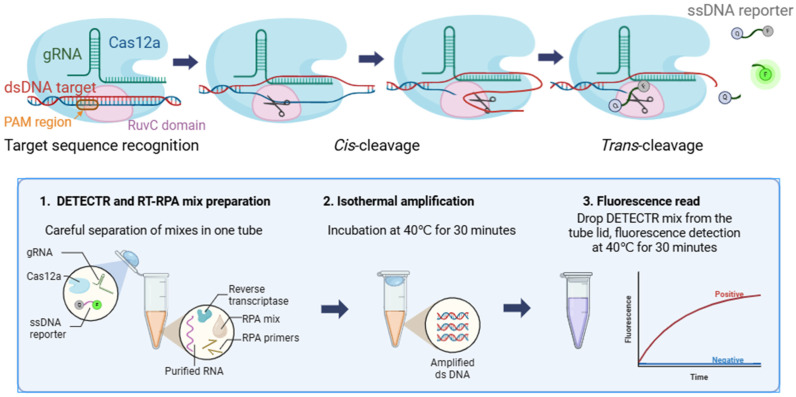
Schematic illustration of the DETECTR method’s principal mechanism (top half) and the RT-RPA/DETECTR assay protocol (bottom half).

The ‘DNA endonuclease-targeted CRISPR trans-reporter’ system (DETECTR) and the ‘specific high-sensitivity enzymatic reporter unlocking’ (SHERLOCK) detection system are similar [5]. They are based on collateral nuclease activity of type V CRISPR effector proteins (AsCas12a, FnCas12a, AaCas12b, LbCas12a) in the former and on type III, V, and VI CRISPR-Cas13 effectors (LwCas13a, CcaCas13b, PsmCas13b, LwaCas13a) in the latter [6,7]. The mechanism of this activity was first demonstrated and explained in 2018 [6]. The DETECTR system is shown schematically in Figure 1.

DETECTR is adjustable for any dsDNA sequence, and the specificity is set by gRNA. The target requires a protospacer-adjacent motif (PAM) sequence in the strand that opposes the gRNA complementary part. For LbCas12a, the PAM sequence is TTTN or its variations [8].

A published study on the fundamental properties of different CRISPR Cas-based diagnostic systems, such as the calculation of enzyme kinetics and catalytic efficiencies of cis- and trans-cleavages, found some critical limitations [9]. Limit of detection values were estimated to be in the range from approximately 0.05 to 100 pM (≈3 × 10^7^ to 6 × 10^8^ copies/mL). This level of sensitivity is sometimes not enough to detect nucleic acids in real clinical samples, so usually an additional pre-amplification step is necessary for DETECTR or SHERLOCK systems. As point-of-care (POC) testing has become more available, different isothermal nucleic acid amplification methods have been combined with CRISPR diagnostics: loop-mediated isothermal amplification (LAMP) [10,11], recombinase polymerase amplification (RPA) [12], and nucleic acid sequence-based amplification (NASBA) [13]. The DETECTR method has already been successfully applied for SARS-CoV-2 [14,15], hepatitis B virus [16], *Cryptosporidium parvum* [17]*, Aphelenchoides besseyi* [18], *Toxoplasma gondii* [19], and *Haemophilus parasuis* detection [20].

In this paper, we aimed to develop a rapid isothermal point-of-care MACV detection assay and conduct a comprehensive evaluation of its analytical performance, including its detection limit, response to nucleotide substitutions, and performance with heterologous samples. The DETECTR method is known in the literature as a promising isothermal “alternative” to conventional real-time RT-PCR. A key aspect of this work was to critically assess this claim by systematically comparing our results with those obtained by other published methods. Furthermore, we specifically probed the hypothesis that such newer developments could potentially replace PCR in point-of-care diagnostic scenarios.

## 2. Results

### 2.1. Target Sequence Selection

The RNA-dependent RNA polymerase gene, located on the L segment, is considered the most conserved part of the MACV genome. Nineteen available *M. machupoense* complete L segment sequences (as of 2025/01/09) were aligned using Mega v.11 software (Figure S1). A 136-nucleotide length fragment was chosen as a model target sequence corresponding to positions 1334 to 1469 in the reference sequence (GenBank NC_005079.1).

### 2.2. Design and Optimization of the RT-RPA/DETECTR Assay

#### 2.2.1. Guide RNA Selection

To develop a combined system of isothermal amplification and detection (RT-RPA/DETECTR), the DETECTR system was first developed and optimized. For the selected region, depending on PAM sequence location, two single guide RNAs (gRNA_1, gRNA_2) were designed (Table S1) and compared. For gRNA_1, the PAM sequence was TTTC, and for gRNA_2, it was TTTG. The gRNA hairpin structure that binds to Cas12a protein consists of 21 bases. The portion complementary to the target has a length of 23 nucleobases in both cases. The GC-content values for gRNA_1 and gRNA_2 are 43.5% and 47.8%, respectively (Figure S2). The signal in samples with gRNA_1 was 10-fold higher than for gRNA_2 by the 30th minute of analysis (approx. 800 versus 80). Thus, gRNA_1 was more optimal and selected for further work. Ten-fold dilutions of template DNA showed that the analytical limit of detection (LOD) of DETECTR with this gRNA is 10^8^ copies/µL or approximately 0.2 nM (Figure S3). Therefore, the sensitivity of the detection system without the amplification step is unsatisfactory for clinical studies.

#### 2.2.2. Primer Selection

Recombinase polymerase amplification (RPA) runs at the same temperature as DETECTR (37–42 °C). For that reason, it was chosen for the combined amplification/detection diagnostic system. For the target region, different combinations of forward and reverse primer variants were screened using electrophoresis of amplification products. By comparing the brightness of product bands, we made an indirect evaluation of primer set efficiency (Figure S4). It was found that the most optimal primer pair was 1F/4R (Table S1), which was used further in the work.

#### 2.2.3. RPA/DETECTR Assay

Based on established protocols [12,21], a method combining DETECTR and RPA in one tube (the RPA/DETECTR assay), applying the selected primer set and gRNA, was developed. It involves careful preliminary physical separation of the two mixtures (amplification, detection) in a single tube (Figure 1).

**Figure 2:**
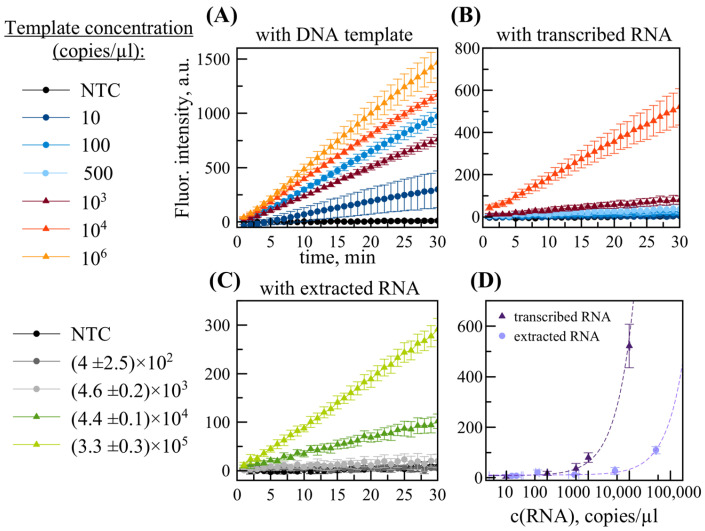
Fluorescence kinetics of the RT-RPA/DETECTR assay at different concentrations of: (**A**) DNA positive control template, (**B**) transcribed RNA positive control, (**C**) RNA extracted from ARP. Panel (**D**) shows the dependence of fluorescence intensity on spiked RNA concentration with approximated curved fitting. The data is presented as the mean of three replicate samples with standard deviation.

Like DETECTR, RPA utilizes a DNA template for reaction. Accordingly, an initial RPA/DETECTR-based analysis was conducted using a positive control DNA template containing a sequence fragment homologous to the viral genome. A series of DNA template dilutions was made, and it showed that the detection limit of the RPA/DETECTR method is 10 copies per reaction (16 aM), as shown in Figure 2a. When the system was tested without preliminary separation of RPA and DETECTR mixes, samples containing the target sequence did not differ from NTC (no-template control), meaning that the RPA/DETECTR system was not working.

The selectivity of the RPA/DETECTR system was tested on sequences of viruses that cause other hemorrhagic fevers: *Mammarenavirus guanaritoense* (GTOV), *Mammarenavirus brazilense* (SABV), *Mammarenavirus juninense* (JUNV), *Henipavirus hendraense* (HENV), and *Henipavirus nipahense* (NPV). The analyses revealed a significant increase in fluorescent signal only in samples containing *M. machupoense* fragments (Figure S5).

### 2.3. Limit of Detection

For detection of RNA template, M-MuLV reverse transcriptase (200 U/µL) was added to the RPA mixture, using 0.3 µL per sample. The positive control template was a transcribed RNA (2300 bases in length) that contained the target MACV viral fragment (136 bases in length). The protocol for the RT-RPA/DETECTR assay was the same as for the RPA/DETECTR assay described above. Analysis of serial dilutions of transcribed RNA template established a detection limit of 500 copies per reaction (0.4 fM), as shown in Figure 2b. This is higher than the value for DNA template. This is presumably due to inhibition of the RPA/DETECTR reaction by reverse transcriptase.

Taking into account that the MACV virus is a BSL-4 agent, we used an artificial system, armored RNA particles (ARPs), to test RT-RPA/DETECTR under conditions close to real clinical tests. The assay gave positive signals when using extracted RNAs with concentrations exceeding (4.6 ± 0.2) × 10^3^ copies per reaction (Figure 2c). This corresponds to approximately 2 × 10^6^ ARP copies/mL.

The dependence of fluorescence on spiked RNA concentration was determined for two sample types (transcribed RNA target sequence, RNA extracted from ARPs) as shown in Figure 2d. Extracted RNA concentration (ddPCR measurement) was calibrated by linear fitting as shown in Figure S6. Concentration dependences were approximated by the linear functions *y(x)* = 0.0549*x* + 8.3566 (R^2^ = 0.9949) and *y(x)* = 0.0022*x* + 10.9845 (R^2^ = 0.9849), respectively (Figure 2d and Figure S7). By calculation, the LOD of the RT-RPA/DETECTR method was estimated to be 450 copies per reaction of transcribed RNA or 10^7^ ARP copies/mL

### 2.4. Impact of Nucleotide Substitutions

A common challenge in developing diagnostics for viral infections is the selection of a conserved genomic region. In some cases, the virus may exhibit high variability (e.g., Lassa virus), wherein no sufficiently conserved sequence of adequate length can be found for PCR primers or fluorescent probes. The region selected for this study is relatively conserved. It is identical for Carvallo strain sequences (AY619642.1, JN794583.1, KM198593.1, AY216511.2, MT015969.1) and some isolates (KU978786.1, KU978789.1).

The selected region has one nucleotide substitution (135/136, 99% identity) in the Chicava strain (AY624354.1, KU978785.1) and isolate KU978790.1. It has two substitutions (134/136, 99% identity) in isolate KU978805.1. Other sequences feature even more differences: 10 substitutions (93% identity) in the Mallele strain (AY619644.1, JN794585.1); 12 substitutions (91% identity) in isolate KU978784.1; 14 substitutions (90% identity) in KU978787.1 and KU978791.1; and 16 nucleotide substitutions (88% identity) in isolate KU978788.1.

**Figure 3:**
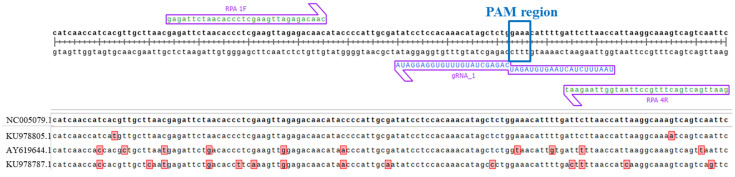
MACV target sequence (NC_005079.1) showing the locations of RPA primers (RPA 1F, RPA 4R) colored in green and gRNA_1 (including PAM region) colored in blue. The aligned MACV sequences (KU978805.1, AY619644.1, KU978787.1) featuring nucleotide substitutions (highlighted in red) are shown below.

Here, in addition to the model target sequence (NC_005079.1), we chose sequences with 2, 10, and 14 substitutions in order to evaluate the influence of mismatches within assay components (primers, gRNA) on analytical performance. These mismatches were designated (Table S2) as mism_2, mism_10, and mism_14. Their corresponding identifiers are KU978805.1 (mism_2), AY619644.1 (mism_10), and KU978787.1 (mism_14). Figure 3 shows the primers, gRNA hybridization site, and alignment of the three chosen sequences (with substitutions indicated).

First, we checked how nucleotide substitutions in the DNA template influence the DETECTR assay. The mism_2 template is identical to the original MACV in the guide RNA region. The mism_10 template contains one substitution in the PAM region, changing it from TTTG to TTAG. One article [8] reports that for the LbCas12a nuclease, the new PAM sequence TTAA is as effective as the classical TTTA, so we expected that the change would not be crucial. The mism_14 template has only one substitution in the region of hybridization between gRNA and dsDNA. We diluted each DNA template to specific concentrations (10^11^, 10^10^, 10^9^, 10^8^ copies/µL and performed DETECTR assays with each. Figure S8 shows that the fluorescent signal kinetics are approximately equal for all template types. The detection limit without an amplification step also remains the same: 10^8^ copies/µL

Next, we investigated the impact of single-nucleotide substitutions on the efficiency of RPA. With mism_2, there is only one substitution, located in the middle of the reverse primer. The mism_10 template contains two point mismatches in each primer. With mism_14, both primers feature four substitutions, with two mismatches in the reverse primer being positioned closely to each other and near the 3′-end, potentially hindering effective hybridization with the template.

Each DNA template was added to the RPA assay (utilizing the RPA basic kit) at concentrations of 10^2^ and 10^4^ copies/µL for estimation of amplification efficiency. The quantity of amplification was evaluated indirectly by brightness of the product bands (136 bp) observed by gel electrophoresis. As shown in Figure S9, for the mism_10 template at a concentration of 10^2^ copies/µL, amplification occurs less intensively compared to the original MACV template and mism_2. However, at a concentration of 10^4^ copies/µL, a product is still generated. This suggests lower reaction efficiency for the mism_10 template, potentially leading to a higher detection limit (lower sensitivity) for the whole system. With mism_14 (both concentrations), electrophoretic visualization indicated minimal difference from the negative control, with no bright band corresponding to the expected amplicon size (136 bp). Thus, these RPA primers appear to be ineffective for detecting templates with a high number of substitutions in the region the assay detects.

**Figure 4:**
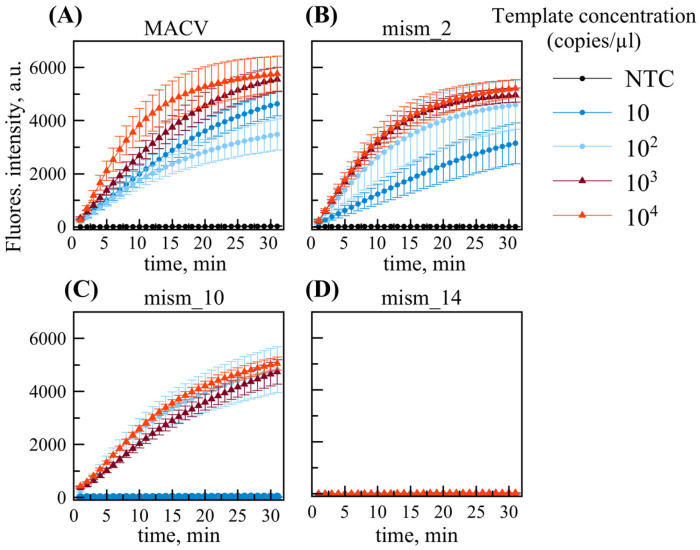
RPA/DETECTR assay with templates at different concentrations using primers RPA 1F and RPA 4R: (**A**) MACV, (**B**) mism_2, (**C**) mism_10, (**D**) mism_14. The data are presented as the average of three replicate samples with standard deviation.

We tested the combined RPA/DETECTR in a single tube using the RPA basic kit. All DNA templates, including the original reference sequence without substitutions, were diluted into a range (10^1^ to 10^4^ copies/µL) using 10-fold serial dilutions (Figure 4). For the MACV templates without substitution, and those with two substitutions, analysis showed a positive result at template concentrations exceeding 10 copies per reaction, thus maintaining the LOD. In contrast, mism_10 exhibited a negative result for 10 copies per reaction, whereas 100 copies were detected positively. Therefore, with the template featuring 10 sequence substitutions, including several affecting assay reagents (2 substitutions in each primer, 1 substitution in the guide RNA), sensitivity decreases (LOD rises to 100 copies/µL). With mism_14, the signal remains negative at all concentrations tested. We assume that the reason is ineffective RPA with these primers.

### 2.5. Multiplex RPA/DETECTR Assay for Detection of MACV Genetic Variants

Following a similar approach used in real-time PCR, we selected modified RPA primer variants (RPA 1F_v2, RPA 4R_v2), each containing four single-nucleotide substitutions at positions corresponding to the mism_14 template. Initial RPA assays were performed with subsequent analysis of amplification products by gel electrophoresis, following the same protocol as in Figure S9. The results of the electrophoretic separation of RPA products with only this primer pair show specific amplification of the mism_14 template, with faint bands visible for mism_10 at a concentration of 10^4^ copies/ µL (Figure S10).

Subsequently, RPA was performed using a mixture of two primer pairs at equimolar concentrations: 1F, 1F_v2, 4R, and 4R_v2 (Figure S11). We used a standard protocol corresponding to 2.4 µL of each 10 µM stock primer per 50 µL reaction. Under these conditions, a bright amplification product band was observed at a concentration of 10^4^ copies/µL for each template variant, with fainter, but still detectable, bands at 10^2^ copies/µL.

Similar results were observed when testing primers with substitutions (Figure S12) and the mixture of all primers (Figure S13) in the RPA/DETECTR assay. Comparative analysis revealed that primers 1F_v2 and 4R_v2 alone efficiently amplify the mism_14 template, with sensitivity below 10 copies per reaction (Figure S12). Primer mixtures enabled the combined RPA/DETECTR system to detect all of the examined genetic variants (MACV target sequences) while maintaining a detection limit below 10 copies/µL for DNA templates (Figure S13).

**Figure 5:**
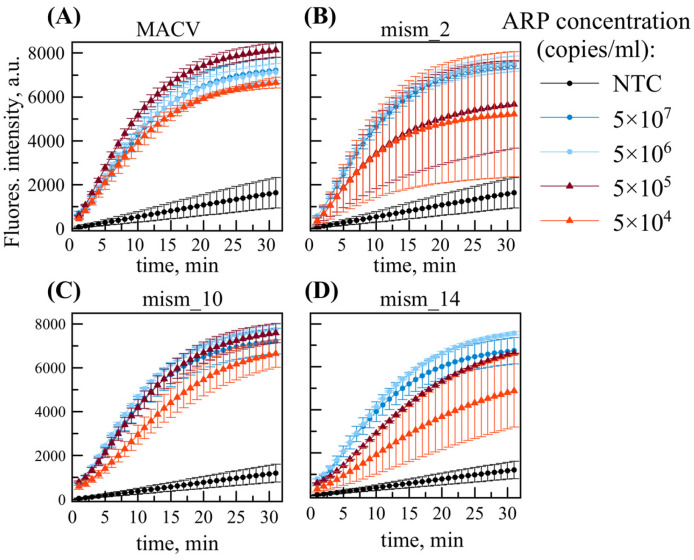
RT-RPA/DETECTR assay using a primer mix and RNAs extracted from ARPs containing: (**A**) MACV, (**B**) mism_2, (**C**) mism_10, and (**D**) mism_14 sequence. The data are presented as the average of three sample replicates with standard deviation.

Notably, primer mixtures were found to increase background fluorescence, as evidenced by increasing signals in no-template controls (NTCs) as seen in Figure S13 and Figure 5. However, the fluorescence intensity of NTCs remained consistently lower than all target-containing samples across tested concentrations.

### 2.6. Multiplex RT-RPA/DETECTR for Virus-like ARPs

The system utilizing the RPA primer mixture was subsequently examined using virus-like ARPs containing MACV, mism_2, mism_10, or mism_14 sequences. Figure 5 shows RT-RPA/DETECTR assay performance using RNAs isolated from virus-like ARPs with a primer mixture (1F, 4R, 1F_v2, 4R_v2). Based on prior ddPCR quantification, input RNA concentrations were precisely adjusted to 10–10^4^ copies per reaction, corresponding to 5 × (10^3^–10^7^) ARP copies/mL.

The primer mixture combined with TwistAmp^®^ Basic (replacing TwistAmp^®^ Basic Liquid, used previously) kit reagents decreased the LOD for MACV template by at least 10-fold (Figure 5a). However, greater signal variability was observed for mism_2 template at low concentration: one sample replicate of three was close to the negative control at the endpoint of analysis (5 × 10^4^ copies/mL red plots in Figure 5b). Therefore, defining LOD as the lowest concentration at which 100% of replicates test positive, despite rising background in NTCs, the system maintained a detection limit of 5 × 10^4^ ARP copies/ mL (≈80 aM), or 100 RNA copies/reaction, across all examined *Mammarenavirus machupoense* genetic variants.

### 2.7. Clinical Validation of RT-RPA/DETECTR

Insofar as MACV is a BSL-4 agent, we validated the developed method by spiking whole blood and serum samples obtained from healthy donors with ARPs. Each biological group contained 5 negative (not spiked) and 5 positive (spiked) samples. For the RT-RPA/DETECTR assay, all sample analyses were performed in duplicate. Comparison between biological samples and controls (water spiked with ARPs) showed that there were no false-positive or false-negative reactions (Figure S14). This indicates that an absence of interference or inhibition from the components present (human nucleic acid, blood, serum). Analysis of the serial dilutions (serum samples spiked with ARPs) showed that the LOD was maintained (Figure S15).

## 3. Discussion

The expansion of grain transportation in Bolivian areas, as well as an increase in exports, may raise the risk that MACV will leave its endemic region and become an international threat. Epidemiological monitoring and early diagnostics usually require the development of rapid, accurate, and inexpensive tests. In countries such as Bolivia that do not have many fully equipped laboratories in endemic regions, methods that do not need special equipment are especially valuable. Here, we developed a new test for MACV based on the CRISPR Cas detection method combined with isothermal amplification in one tube. A 2021 critical review, as well as many articles on similar topics, calls these molecular diagnostic systems a promising, accurate, and sensitive approach [7]. We would like, however, to discuss whether such statements might be somewhat controversial or overly ambitious.

The DETECTR and SHERLOCK systems clearly have numerous advantages: they are less time-consuming; they do not require a thermocycler; and single-tube assays carry a decreased risk of contamination. In addition, it has been shown that a simple portable LED device can be used for RPA-based CRISPR Cas12 diagnostics, where fluorescence signals are captured by a cell phone, or even by the naked eye [12]. This makes such tests even more affordable. Another advantage of the DETECTR assay is that there are options for non-fluorescent detection by lateral-flow analysis. This eliminates the requirement for fluorescence reading equipment completely. However, it needs to be taken into account that studies usually show that lateral-flow assays are less sensitive than fluorescent methods [17,18,22].

Hence, we compared our developed test to similar detection systems (for various targets) presented in other articles. Table 1 shows the summary results of this comparison by target and limit of detection, recalculated to the same units (copies/mL). A direct comparison shows that the RT-RPA/DETECTR detection system developed in this work features a real limit of detection of approximately 100 RNA copies per reaction or 5 × 10^4^ copies/mL of virus-like ARPs (80 aM). This means that DETECTR-RPA, or analogous assays, are strongly dependent on target sequence selection. The current study, and comparison with other articles in Table 1, indicates that LOD calculation is a nuanced topic. LOD usually depends on the choice of sample material to be tested and on the method of target concentration quantification. The latter often cannot be validated for many real clinical samples. Rare pathogens in particular, or even the recently widespread SARS-CoV-2, can have this measurement validation problem. Other comprehensive reviews of CRISPR-based diagnostic platforms for animal infections [23,24] have revealed a wide range of estimated limits of detection. These range from 1000 pM (≈6 × 10^11^ copies/mL) down to single copy detection.

Another general drawback of CRISPR-based diagnostics noted elsewhere [26] is that these methods can be applied only to qualitative studies. The RT-RPA/DETECTR assay described here is based on fluorescence intensity analysis. Unlike Ct values in real-time RT-PCR, the results are relative. Target concentrations in real-time RT-PCR can be quantified using a calibration curve (qPCR) or measured precisely by droplet digital PCR.

Other works from Table 1 also show that it is possible to improve the analytical sensitivity of RT-RPA/DETECTR by several means. These include selection of a more effective target region for detection; more careful selection of primers; and careful adjustment of reaction conditions and composition (i.e., replacement of reverse transcriptase). Nevertheless, LOD improvement does not solve the deeper problem of this method: the DETECTR and SHERLOCK systems have fundamental limitations in sensitivity [9]. As such, an amplification step is still required, in this case using RPA, or in other works, using LAMP. In some cases, it may be more logical (effective, easier, cheaper) to use RPA or LAMP amplification techniques with other detection means (fluorescent probes, SYBR dyes, lateral-flow analysis, colorimetry) instead of making the system more complicated.

## 4. Materials and Methods

All DNA primers and probes listed in Table S1 were purchased from DNA Synthesis (Russia), while gRNAs were obtained from GenTerra (Moscow, Russia). They were dissolved in ultrapure water (Simplicity UV, Millipore, Merck, Darmstadt, Germany) and stored at −20 °C before use. Fluorescent probes and gRNAs were aliquoted and were not refrozen or defrosted more than once. Graphical representations of data were made in MagicPlot 3.0.1.0 software (Magicplot Systems, LLC, Saint Petersburg, Russia).

### 4.1. Positive Control Recombinant Plasmid Construction

Target MACV sequence was synthesized de novo by the PCR assembly method, as previously described [27]. Briefly, a set of overlapping oligonucleotides was synthesized, and the reaction mixture contained 30 pmol of outer oligonucleotides (MACV_1, MACV_4) and 1 pmol of inner oligonucleotides (MACV_2, MACV_3). DNA assembly was performed with Phusion High-Fidelity DNA Polymerase (New England Biolabs, Ipswich, MA, USA). The reaction was prepared according to the manufacturer’s protocol, and the following thermal program was used: 30 sec at 98 °C; followed by 27 cycles (10 sec at 98 °C, 30 sec at 62 °C, 10 sec at 72 °C); and a final extension step (5 min at 72 °C). The PCR product was purified by the DNA Clean & Concentrator Kit (Zymo Research, Tustin, CA, USA), A-tailed with Taq DNA Polymerase (Evrogen, Moscow, Russia), ligated into the pGEM–T plasmid vector (Promega, Madison, WI, USA), and transformed into NEB Turbo *E. coli* (New England Biolabs, Ipswich, MA, USA). The correctness of the assembly was verified by Sanger sequencing.

After verification, plasmids from individual bacterial clones were purified using the Plasmid Miniprep Kit (Zymo Research, Tustin, CA, USA). The target DNA fragment was then amplified by TaqM Master Mix (Intifica, Saint Petersburg, Russia) using primers MACV_1 and MACV_4. The amplicon was purified and cloned into a linearized in–house pET-MS2 plasmid vector and transformed into NEB Turbo *E. coli* (New England Biolabs, Ipswich, MA, USA). After confirming its correctness by DNA sequencing, the recombinant plasmid was purified using the Plasmid Miniprep Kit (Zymo Research, Tustin, CA, USA).

Target sequences with nucleotide substitutions (mism_2, mism_10, mism_14) as shown in Table S2, and corresponding recombinant plasmids, were synthesized using oligonucleotide sets. For mism_2, the set was MACV_2_1f/MACV_2_2f. For mism_10, the set was MACV_10_1/MACV_10_2/MACV_10_3/MACV_10_4. For mism_14, the set was MACV_14_1/MACV_14_2/MACV_14_3/MACV_14_3. Negative control plasmids containing GTOV, SABV, JUNV, HENV, and NPV fragments were constructed similarly.

### 4.2. DNA and RNA Positive Controls

Recombinant plasmids were used for PCR to obtain 2300 bp linear regions containing target fragments and flanking sequences. The composition of the PCR mix and the PCR protocol are shown in Table S3. After purification of the amplification products by AMPure XP Bead-Based Reagent (Beckman Coulter, Brea, CA, USA), the concentration of the obtained positive-control DNA was measured using the NanoDrop One^C^ system (Thermo Fisher Scientific, USA). Further, it was diluted to the required concentrations with ultrapure water.

The linear, positive-control DNA was transcribed into RNA using the T7 RiboMAX™ Express Large Scale RNA Production System kit (Promega, Madison, WI, USA) according to the standard protocol. After incubation, 1 µL of DNase was added to the reaction. It was followed by similar purification using AMPure XP Bead-Based Reagent (Beckman Coulter, Brea, CA, USA) and measurement of the transcription product using the NanoDrop One^C^ (Thermo Fisher Scientific, Waltham, MA, USA). Purified positive-control RNA was diluted to the required concentrations for further analysis in RNA buffer from the RIBO-prep kit (AmpliSens, Moscow, Russia).

### 4.3. Production and Purification of Armored RNA Particles (ARP)

The recombinant plasmid was transformed into an *E. coli* BL-21 (DE3) strain, and protein expression was induced with 1 mM isopropyl-L-thio-D-galactopyranoside (IPTG, neoFroxx, Einhausen, Germany) at 37 °C for 4 h. Cells were collected by centrifugation, lysed with lysozyme and freeze—thawing, and treated with DNase I (Zymo Research, Tustin, CA, USA) and RNase A (Zymo Research, Tustin, CA, USA). Armored RNA particles (ARP) in the supernatant were purified by CsCl (PanReac AppliChem, Chicago, IL, USA) density gradient centrifugation (200,000× *g* for 22 h at 20 °C). Positive control ARPs from three main fractions were tested by real-time RT-PCR (Figure S15) using specific oligonucleotides (MACV_F, MACV_R, MACV_probe) listed in Table S1. The third fraction was chosen for further use based on the lowest Ct value.

### 4.4. RNA Extraction, Purification, and Droplet Digital PCR

Armored RNA particles were diluted by a series of 10-fold dilutions (10^−1^ to 10^−7^) from the initial concentration. All samples were handled as three replicates. RNA was isolated from all samples using the RIBO-prep Nucleic Acid Isolation and Purification Kit (AmpliSens, Moscow, Russia) according to the standard protocol. Freshly isolated and purified RNA was used immediately for analysis to avoid degradation. To determine RNA concentrations, droplet digital PCR (ddPCR) was performed. We used the One-Step RT-ddPCR Advanced Kit for Probes (Bio-Rad, Hercules, CA, USA) and prepared reaction mixes according to the standard protocol (Table S4). Probe (SK_probe) and primers (SK_F, SK_R) were designed for an artificial insert in a flanking sequence [28]. All samples contained more than 10,000 accepted droplets. A threshold was manually set higher than the no-template control (NTC) sample level.

We calculated the initial RNA concentration, averaged the results for 3 identical samples, and determined the linear correlation (log_10_ scale) between the calculated number of copies and the RNA dilution (Figure S2). Certain samples were excluded from analysis due to being outside of the reliable range of ddPCR: undiluted RNA and specific dilutions (10^−1^, 10^−2^, 10^−7^).

### 4.5. DETECTR Assay

The ssDNA fluorescent reporter for the DETECTR assay was selected based on well-established sequences reported in the literature [6,8,12,14]. Positive control DNA template was diluted to 10^10^, 10^9^, and 10^8^ copies/µL. The preparation of DETECTR mix is shown in Table S5. All reagents were added to 200 µL PCR tubes with optical caps. Handling was performed using a refrigerated block PCR Cooler (Eppendorf, Hamburg, Germany), and samples were thoroughly mixed after addition. Immediately after the addition of the template, samples were placed in the CFX96 Touch (Bio-Rad, Hercules, CA, USA) for detection on the FAM channel at 40 °C for 30 min, with plate readings every 1 min.

### 4.6. RPA Assay

RPA primers were manually designed according to the manufacturer’s recommendations (TwistAmp manual, TwistDx, Maidenhead, UK). Screening of RPA primers was performed using TwistAmp^®^ Liquid Basic kit reagents (TwistDx™, Maidenhead, UK) as follows. The reaction mixture was prepared by the standard kit protocol with component volumes adjusted (Table S6) for the total reaction volume (15 )µL. Samples were incubated in a TDB-120 thermostat (BioSan, Riga, Latvia) at 40 °C for 30 min.

Agarose (Helicon, Moscow, Russia) gels were prepared containing 1× TBE buffer with 89 mM Tris (Helicon, Moscow, Russia), 89 mM H_3_BO_3_ (Rosmedbio, Russia), and 2 mM Na_2_EDTA (PanReac AppliChem, Chicago, IL, USA). After amplification, RPA products (5 )µL were mixed with 1 µL of 6x Purple Gel Loading Dye (New England Biolabs, Ipswich, MA, USA). Samples were loaded onto 2% agarose gels in 1x TBE buffer (pH 8.0), stained with 1% Ethidium Bromide (PanReac AppliChem, Chicago, IL, USA). The concentration of DNA template was 10^8^ copies/µL. TriDye™ Ultra Low Range DNA Ladder (New England Biolabs, Ipswich, MA, USA) was loaded (2 )µL into side wells (10 to 700 bp range). Electrophoresis was carried out in the Mini-Sub Cell GT (Bio-Rad, Hercules, CA, USA) at 80 V for 75 min. Gels were visualized using the gelLITE Gel Documentation System (Cleaver Scientific, Rugby, UK). The most optimal primer pair was selected based on the intensity of the specific amplification bands observed by gel electrophoresis (Figure S4).

Testing of templates with nucleotide substitutions was conducted using the TwistAmp^®^ Basic kit (TwistDx™, Maidenhead, UK). Tubes containing lyophilized reagents were rehydrated by Primer Free Rehydration buffer, primer mix, and water according to manufacturer instructions. Subsequent procedures were performed similarly to those described above for the TwistAmp^®^ Liquid Basic kit.

### 4.7. Single-Tube RT-RPA/DETECTR

For 25 µL of RT-RPA/DETECTR single-tube reaction, 14 µL of RT-RPA mix and 10 µL of DETECTR mix were prepared (Table S7). Using a cooling block, the DETECTR mix was carefully added to the inner surface of the lid of a 200 µL PCR tube with an optical cap. The RT-RPA mix was carefully added to the bottom of the tube. We quickly added magnesium acetate and 20x Core mix to the RT-RPA master mix and vortexed right before adding template. One microliter of RNA was added to the RT-RPA mix, thoroughly pipetted (in place), and the tube was carefully closed such that the DETECTR mix did not fall from the cap. Samples were then incubated at 40 °C for 30 min. After isothermal amplification with reverse transcription completed, tubes were spun on a minicentrifuge-vortex device (Microspin FV-2400, BioSan, Riga, Latvia) to pull the DETECTR mix down from the lid. The total (combined) reaction mixture was vortexed briefly, and tubes were placed in the CFX96 Touch device (Bio-Rad, Hercules, CA, USA) with the same protocol used for DETECTR analysis: 30 min at 40 °C with plate reads every 1 min (FAM channel).

### 4.8. Limit of Detection (LOD) Estimation

In this work, we define analytical LOD as the minimum concentration of target sequence that provides a positive result in 100% of sample replicates. Unless otherwise indicated, fluorescent signals for samples are always higher than signals for no-template controls (NTC). LOD was calculated by the common definition: *LOD = ((blank + 3.2S − b))/a*, where *a* and *b* are coefficients in the *y(x) = ax + b* dependence of fluorescence signal *y(x)* on target concentration *x*; *blank* is NTC fluorescence; and *S* is NTC standard deviation.

### 4.9. Clinical Validation

The RT-RPA/DETECTR method was validated on 2 biological sample groups: whole blood and serum samples obtained from healthy donors. Each biological group contained 5 different negative samples and 5 positive samples. Positive samples were prepared by spiking the biological matrix with virus-like ARPs using the following procedure: 10 µL of ARP solution was added to 90 µL of serum or whole blood to create the initial spiked stock. Serial tenfold dilutions were then prepared in the respective biological matrix (10 µL of previous dilution + 90 µL of fresh matrix) to achieve the desired concentrations. These spiked biological samples were compared to control samples prepared by spiking water with ARPs in the same manner.

All experiments involving human serum and whole blood samples were conducted in accordance with the principles of the Declaration of Helsinki and approved by the Institutional Review Board of the Saint Petersburg Pasteur Institute (St. Petersburg, Russia, No. 065-10, approval date 5 July 2025). Verbal informed consent was obtained from all participants prior to blood collection. The use of verbal consent was specified in the study protocol approved by the Institutional Review Board of the Saint Petersburg Pasteur Institute. All samples were fully anonymized prior to analysis.

## 5. Conclusions

Here, we report the development of a real-time fluorescent method for *M. machupoense* virus detection: the RT-RPA/DETECTR assay. It combines the DETECTR system with nuclease Lba Cas12a and recombinase polymerase amplification in one tube. After optimization, the limits of detection were estimated to be 10 copies per reaction of positive control DNA or about 5 × 10^4^ copies/mL of specially designed virus-like ARPs. The system was tested on templates containing different numbers of nucleotide substitutions. It was also modified by multiplexing primers for different genetic variants of the target sequence.

Our findings provide a definitive answer to our initial hypothesis: while the CRISPR-Cas12a platform represents a significant advancement for rapid, equipment-limited diagnostics, it currently serves as a powerful complementary tool rather than a complete replacement for conventional PCR. This conclusion is supported by the method’s dependency on target sequence selection, its provision of primarily qualitative results, and its current operational complexity compared to established PCR workflows.

For practical implementation, this assay can be recommended for initial screening and outbreak monitoring in resource-limited settings where rapid results are prioritized over absolute quantification. In clinical settings requiring high throughput or precise viral load measurement, traditional RT-PCR remains the gold standard. Future development should focus on creating stable, lyophilized reagent formulations and optimizing multiplex capabilities to enhance the platform’s utility in field-deployable diagnostic scenarios.

## 6. Patents

Patent No. 2832917, Int. Cl. C12Q 1/68 (2024.08); C12Q 1/6876 (2024.08). Method for detecting Mammarenavirus Machupoense by DETECTR method with isothermal amplification. No. 2024103818; application: 14.02.2024; date of publication 10.01.2025/Kapitonova M.A., Shabalina A.V., Dedkov V.G., Dolgova A.S. Proprietors: Federalnoe byudzhetnoe uchrezhdenie nauki «Sankt-Peterburgskij nauchno-issledovatelskij institut epidemiologii i mikrobiologii im. Pastera Federalnoj sluzhby po nadzoru v sfere zashchity prav potrebitelej i blagopoluchiya cheloveka» (FBUN NII epidemiologii i mikrobiologii imeni Pastera) (RU).

## Figures and Tables

**Table 1:** Comparison of published Cas-based detection systems.

Detection System	Target Name, Fragment Length	Test Material	Published LOD	Recalculated LOD	Reference
Cas12a-DETECTR system after pre-amplification by LAMP	Hepatitis B virus polymerase coding region, 188 nt	standard plasmids	1 copy/µl	10^3^ copies/ml	[16]
One-pot RPA-CRISPR/Cas13a assay	Nipah virus (AY988601.1) N gene, 231 nt	cDNA from healthy human plasma mixed with synthetic Nipah virus N gene RNA	10^3^ copies/µl	10^6^ copies/ml	[22]
AIOD-CRISPR assay	SARS-CoV-2 N gene, 316 nt	a positive control plasmid, clinical swab samples	3 copies per reaction with two gRNAs, 3 × 10^3^ copies per reaction with one gRNA	3 × 10^3^ copies/,mL 3 × 10^6^ copies/ml	[14]
DETECTR assay (Cas12 detection with lateral-flow analysis after pre-amplification by RT-LAMP)	SARS-CoV-2 E gene (WHO E amplicon) and N gene (CDC N2 amplicon)	synthetic gene fragments, nasopharyngeal swab	10 copies/ µL	2 × 10^4^ copies/ml	[15]
CRISPR-Cas13a/C2c2 (RT-RPA with T7 RNA polymerase and subsequent SHERLOCK detection assay by Cas13a)	Zika virus, 343 nt	lentiviruses harboring genomic fragments, clinical samples (serum or urine)	2 aM	1.2 × 10^3^ copies/ml	[25]
Multiplex, single-tube RT-RPA/DETECTR assay	*M. machupoense* virus, 136 nt	virus-like ARPs	80 aM	5 × 10^4^ copies/ml	this work

## Data Availability

Data is contained within the article or Supplementary Materials.

## Supplementary Materials

The following supporting information can be downloaded at: https://www.mdpi.com/article/10.3390/ijms26199754/s1, Figure S1: Alignment of M. machupoense virus L segment sequence fragments; Figure S2: Comparison of two guide RNAs (gRNA_1, gRNA_2) by fluorescence intensity in the DETECTR assay; Figure S3: Fluorescence kinetics of the DETECTR assay with different concentrations of positive control DNA; Figure S4: Screening of forward (1F, 2F) and reverse (4R, 5R, 6R) RPA primers by electrophoresis; Figure S5: Selectivity analysis of the RT-RPA/DETECTR method; Figure S6: Dependence and linear fit (Log10 scale) of calculated extracted RNA concentration by ddPCR on initial 10-fold dilutions of ARP samples; Figure S7: RT-RPA/DETECTR assay data linear approximation: mean RFU signal versus concentration of target RNA extracted from ARPs; Figure S8: DETECTR assay fluorescence kinetics. Positive control (MACV) template, and three templates with mismatches (mism_2, mism_10, mism_14), are shown; Figure S9: RPA assay with different templates at two concentrations; Figure S10: RPA assay with different templates at two concentrations with primers for nucleotide substitution detection (RPA 1F_v2, RPA 4R_v2). The higher (10^4^, bottom) and lower (10^2^, top) concentrations (copies/µL) are shown; Figure S11: RPA assay with different templates at two concentrations with a mix of primers for nucleotide substitution detection. Primers used were RPA 1F, RPA 4R, RPA 1F_v2, and RPA 4R_v2; Figure S12: Two-primer RPA/DETECTR assay. Templates MACV, mism_2, mism_10, and mism_14 at different concentrations (10–10^4^ copies/µL) are shown. Primers with nucleotide substitutions (RPA 1F_v2, RPA 4R_v2) were used; Figure S13: Four-primer RPA/DETECTR assay. Templates MACV, mism_2, mism_10, and mism_14 at different concentrations (10–10^4^ copies/µL) are shown. A mix of primers (RPA 1F, RPA 4R, RPA 1F_v2, RPA 4R_v2) was used (each at 1x concentration); Figure S14: (a) RT-RPA/DETECTR assay using primer mix and RNAs extracted from ARP spiked water (control samples), serum, and whole blood samples. (b) Comparison of endpoint fluorescent intensity; Figure S15: Four-primer RPA/DETECTR assay with RNAs extracted from ARP spiked serum samples at different concentrations; Table S1: Primer, fluorescent probe, and gRNA sequences used in this work; Table S2: Target sequences used in this work; Table S3: PCR reaction mix and conditions to obtain a linear DNA positive control; Figure S16: Selection of armored RNA particle (ARP) fraction by real-time RT-PCR; Table S4: Reaction mix and conditions for ddPCR using the One-Step RT-ddPCR Advanced Kit for Probes; Table S5: Reaction mix for the DETECTR assay; Table S6: Reaction mix for the RPA assay using TwistAmp^®^ Liquid Basic kit reagents (TwistDx™, Maidenhead, UK); Table S7: Reaction mixes for the combined single-tube RT-RPA/DETECTR assay. The higher (10^4^, bottom) and lower (10^2^, top) concentrations (copies/µL) are shown.

## Abbreviations

The following abbreviations are used in this manuscript:

BHF	Bolivian hemorrhagic fever
MACV	*Mammarenavirus machupoense*
GTOV	*Mammarenavirus guanaritoense*
SABV	*Mammarenavirus brazilense*
JUNV	*Mammarenavirus juninense*
HENV	*Henipavirus hendraense*
NPV	*Henipavirus nipahense*
BSL-4	Biosafety Level-4
DNA	Deoxyribonucleic acid
dsDNA	double-stranded DNA
RNA	Ribonucleic acid
CRISPR	Clustered regularly interspaced short palindromic repeats
PAM	Protospacer-adjacent motif
DETECTR	DNA endonuclease-targeted CRISPR trans-reporter
SHERLOCK	Specific high-sensitivity enzymatic reporter unlocking
PCR	Polymerase chain reaction
RT-PCR	Reverse transcription polymerase chain reaction
ddPCR	Droplet digital PCR
RPA	Recombinase polymerase amplification
RT-RPA	Reverse transcription recombinase polymerase amplification
LAMP	Loop-mediated isothermal amplification
NASBA	Nuclear acid sequence-based amplification
ELISA	Enzyme-linked immunosorbent assay
POC	Point-of-care
LOD	Limit of detection
NTC	No-template control
ARP	Armored RNA particle
