# Supplementary material for: CRISPR-Cas12a-Based Isothermal Detection of Mammarenavirus machupoense Virus: Optimization and Evaluation of Multiplex Capability
Source: Int J Mol Sci. 2025 Oct 7;26(19):9754. doi: 10.3390/ijms26199754 (PMC12525330; doi:10.3390/ijms26199754)
Supplement: Supplementary file 1 [file ijms-26-09754-s001.zip › ijms-3903861-supplementary.pdf]

Supporting information

CRISPR-Cas12a-Based Isothermal Detection of *M. machupoense* Virus: Optimization and Evaluation of Multiplex Capability

Marina A. Kapitonova [1], Anna V. Shabalina [1], Vladimir G. Dedkov [1,2], and Anna S. Dolgova [1]*

[1] Laboratory of Pathogen Molecular Genetics, St. Petersburg Pasteur Institute, St. Petersburg 197101, Russia

[2] Martsinovsky Institute of Medical Parasitology, Tropical and Vector Borne Diseases, First Moscow State Medical University (Sechenov University), Moscow 119048, Russia

***Correspondence**: annadolgova@inbox.ru, dolgova@pasteurorg.ru

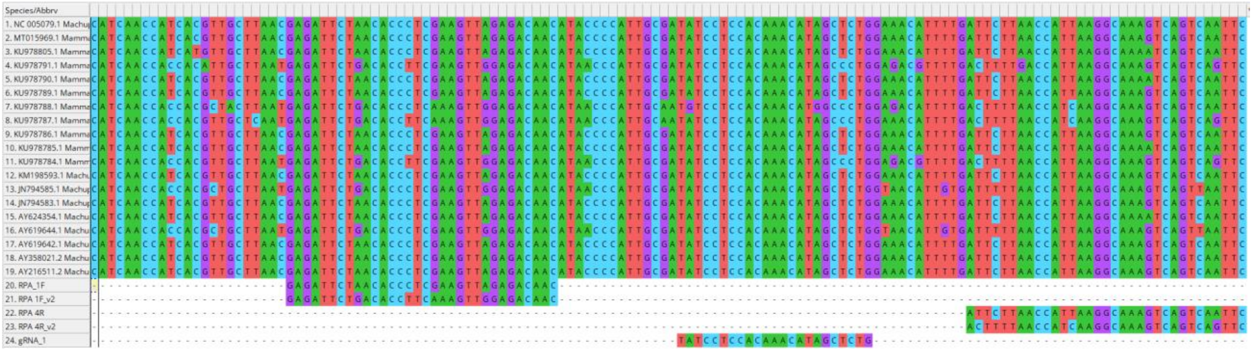

Figure S1. Alignment of *M. machupoense* virus L segment sequence fragments. Sequences with the following GenBank accession numbers are shown: NC_005079.1 (NCBI Reference Sequence), MT015969.1 (strain Carvallo), KU978805.1 (isolate SPB201004275), KU978791.1 (isolate OBT2102), KU978790.1 (isolate Calomys 9301012), KU978789.1 (isolate Calomys 14795), KU978788.1 (isolate 9530537), KU978787.1 (isolate 9430069), KU978786.1 (isolate Calomys 221600), KU978785.1 (isolate 930060_Chicava), KU978784.1 (isolate FSB3270), KM198593.1 (strain Carvallo), JN794585.1 (strain Mallele isolate USAMRIID ID 780), JN794583.1 (strain Carvallo isolate USAMRIID ID 18464), AY624354.1 (strain Chicava), AY619644.1 (strain Mallele), AY619642.1 (strain Carvallo), AY358021.2 (strain Carvallo), and AY216511.2 (strain Carvallo). Also shown are the RPA_1F, RPA 1F_v2, RPA_4R, RPA 4R_v2 primers, and gRNA_1.

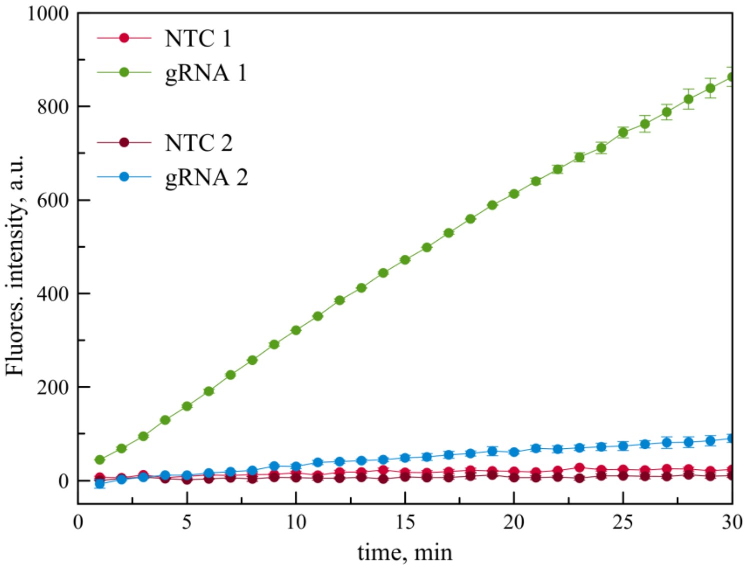

Figure S2. Comparison of two guide RNAs (gRNA_1, gRNA_2) by fluorescence intensity in the DETECTR assay. The same DNA template (10^{10} copies/μl) was used. The data are presented as the average of three sample replicates with standard deviation.

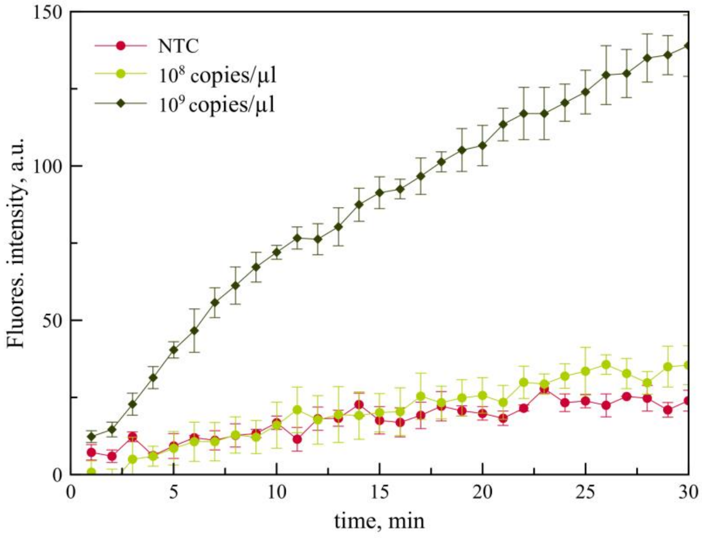

Figure S3. Fluorescence kinetics of the DETECTR assay with different concentrations of positive control DNA. The data are presented as the average of three sample replicates with standard deviation.

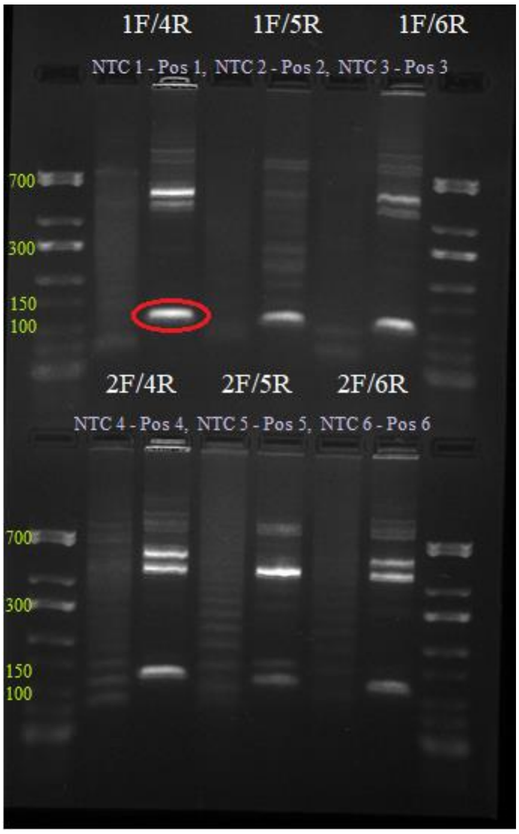

Figure S4. Screening of forward (1F, 2F) and reverse (4R, 5R, 6R) RPA primers. 'Pos 1-6' are positive control RPA reactions with DNA template at 10^6 copies/µl for every primer set. 'NTC 1-6' are no-template negative controls. RPA products are about 105-136 bp in length. The brightest RPA product is indicated in red. Electrophoresis was performed using a 2% agarose TBE gel and a 10-700 nucleotide ladder.

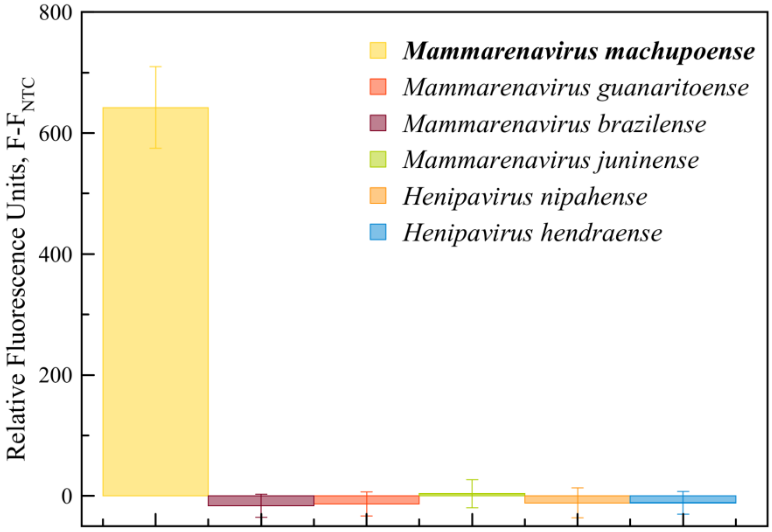

Figure S5. Selectivity analysis of the RT-RPA/DETECTR method. Comparison of fluorescent signal at 30 minutes of analysis with different target sequences is shown. The data are presented as the average of three sample replicates with standard deviation.

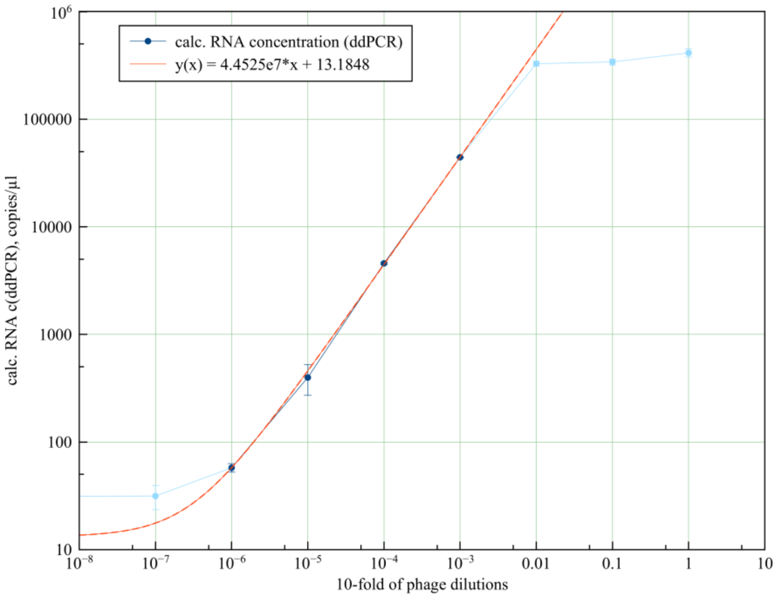

Figure S6. Dependence and linear fit (Log$_{10}$ scale) of calculated extracted RNA concentration by ddPCR on initial 10-fold dilutions of ARP samples.

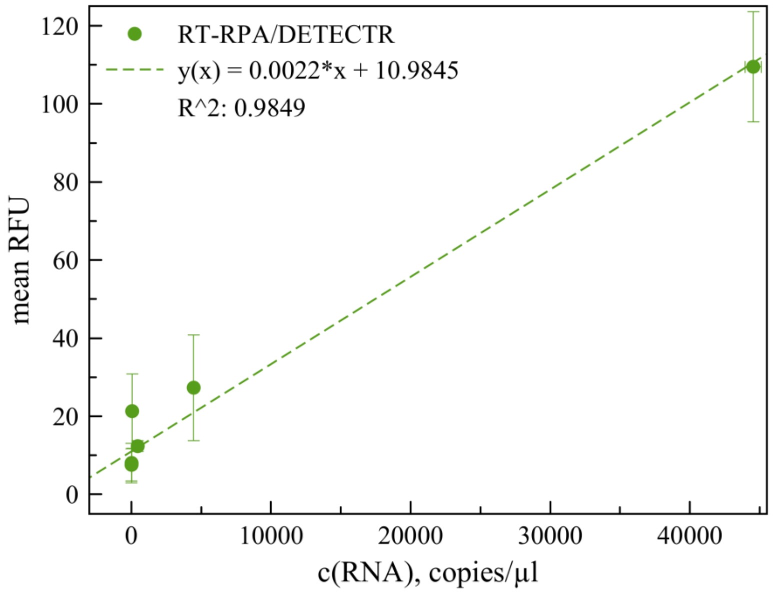

Figure S7. RT-RPA/DETECTR assay data linear approximation: mean RFU signal versus concentration of target RNA extracted from ARPs.

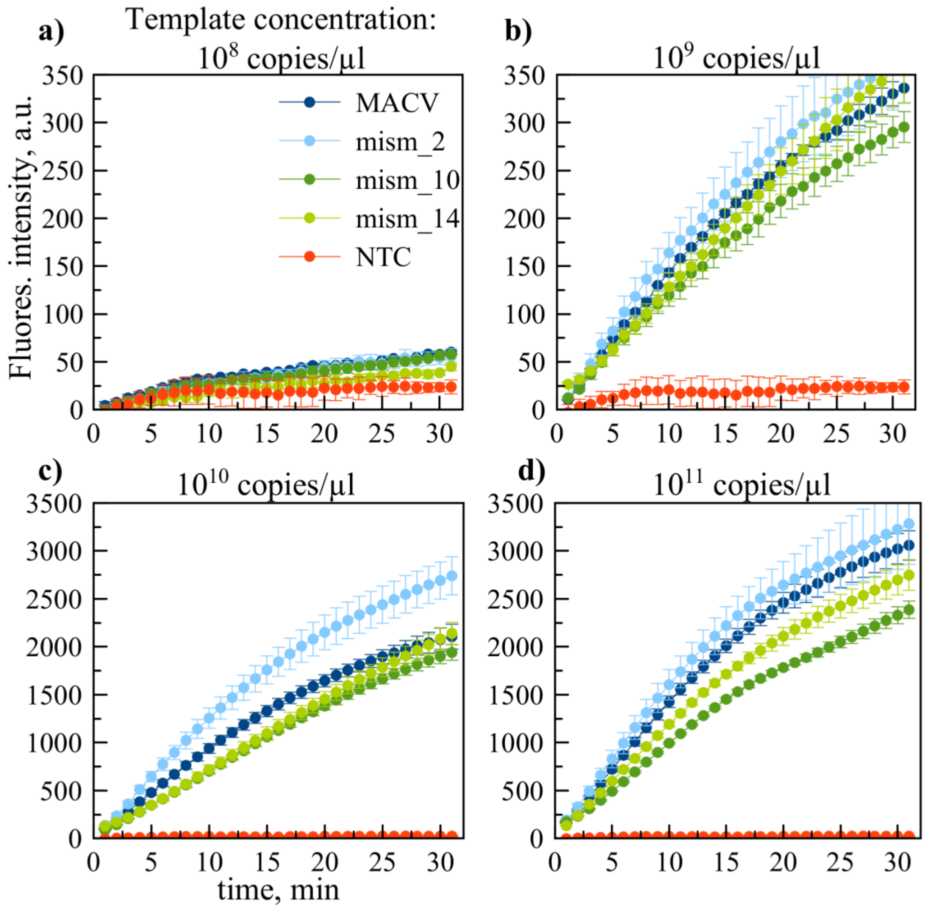

Figure S8. DETECTR assay fluorescence kinetics. Positive control (MACV) template, and three templates with mismatches (mism_2, mism_10, mism_14), are shown. Two concentrations are also indicated. The data are presented as the average of three sample replicates with standard deviation.

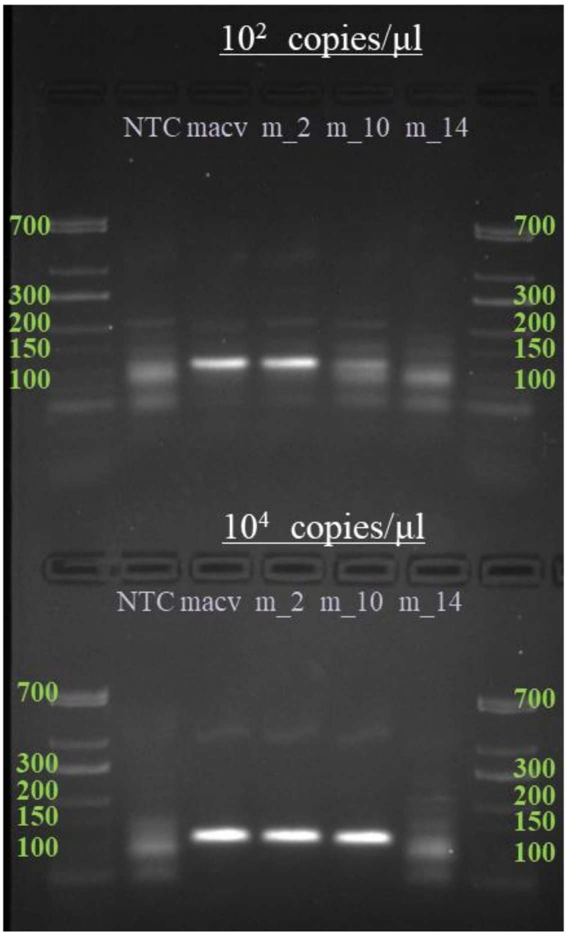

Figure S9. RPA assay with different templates at two concentrations. The higher (10^4, bottom) and lower (10^2, top) concentrations (copies/µl) are shown. Note: NTC – no template control, macv – MACV template, m_2 – mism_2, m_10 – mism_10, m_14 – mism_14.

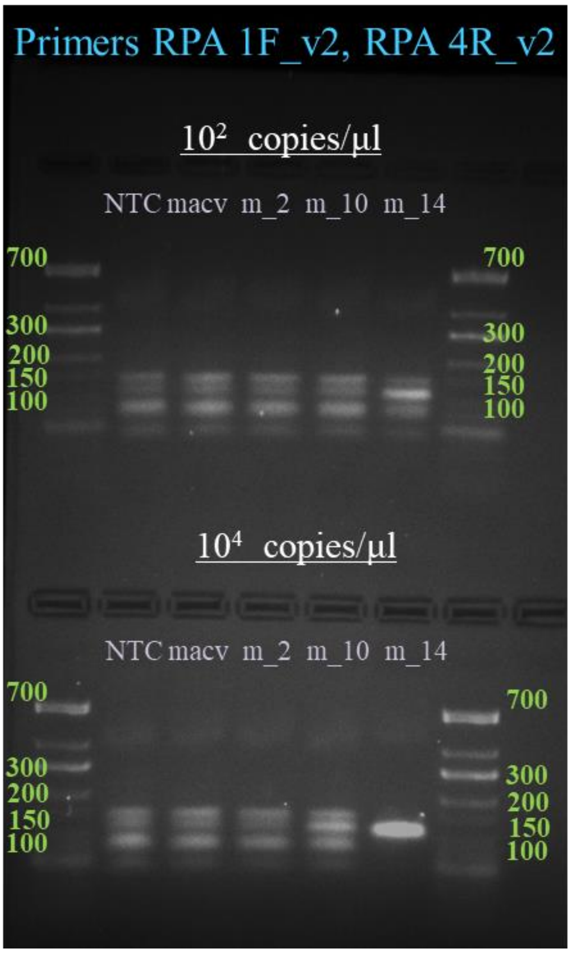

Figure S10. RPA assay with different templates at two concentrations with primers for nucleotide substitution detection (RPA 1F_v2, RPA 4R_v2). The higher (10^4, bottom) and lower (10^2, top) concentrations (copies/µl) are shown. Note: NTC – no template control, macv – MACV template, m_2 – mism_2, m_10 – mism_10, m_14 – mism_14.

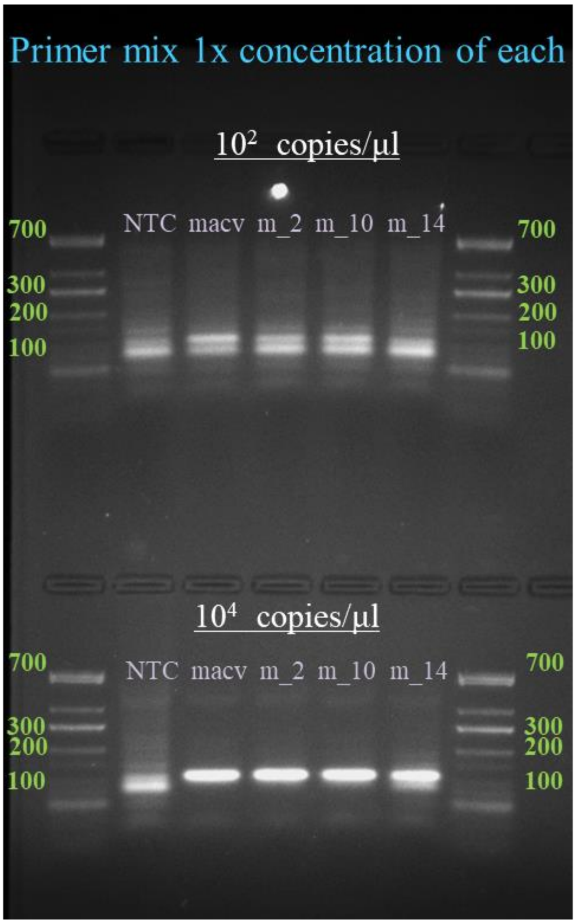

Figure S11. RPA assay with different templates at two concentrations with a mix of primers for nucleotide substitution detection. Primers used were RPA 1F, RPA 4R, RPA 1F_v2, and RPA 4R_v2. The higher (10^4, bottom) and lower (10^2, top) concentrations (copies/µl) are shown. Note: NTC – no template control, macv – MACV template, m_2 – mism_2, m_10 – mism_10, m_14 – mism_14.

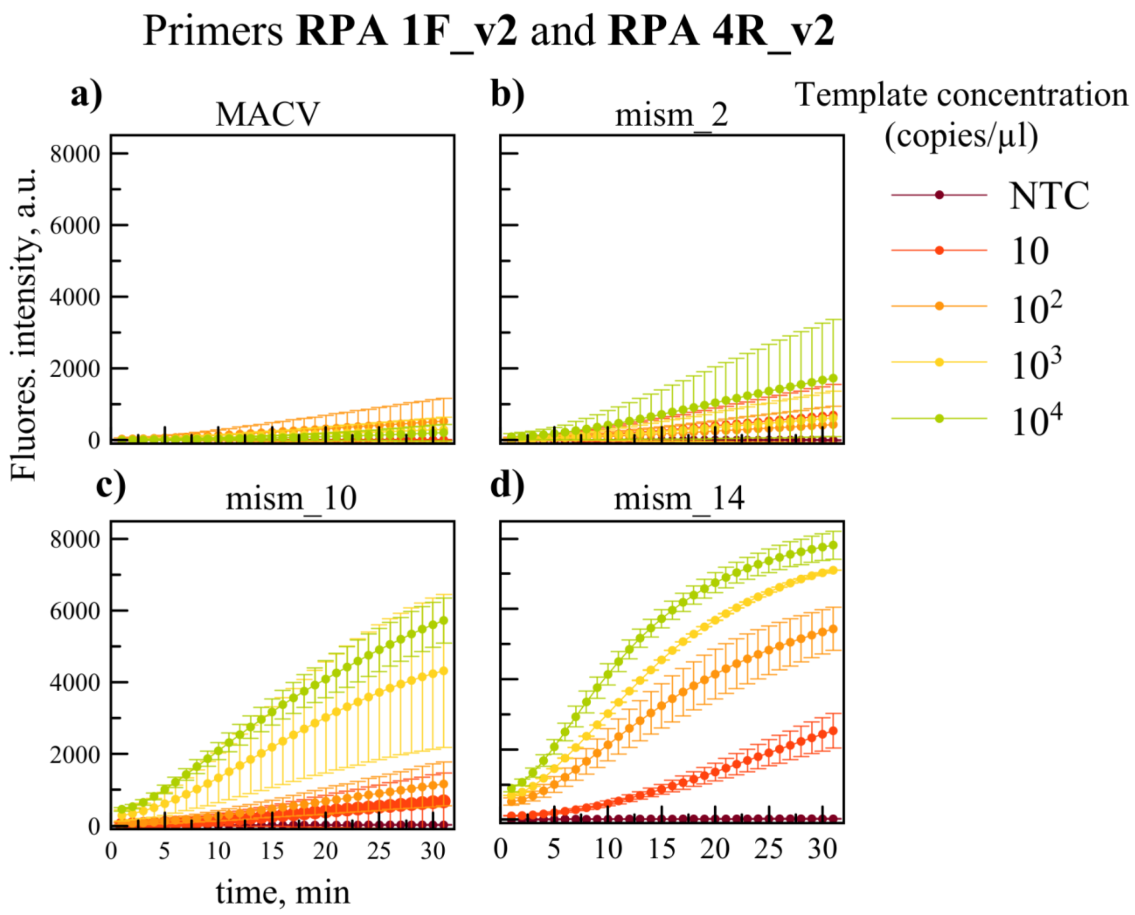

Figure S12. Two-primer RPA/DETECTR assay. Templates MACV, mism_2, mism_10, and mism_14 at different concentrations (10-10^4 copies/µl) are shown. Primers with nucleotide substitutions (RPA 1F_v2, RPA 4R_v2) were used. The data are presented as the average of three sample replicates with standard deviation.

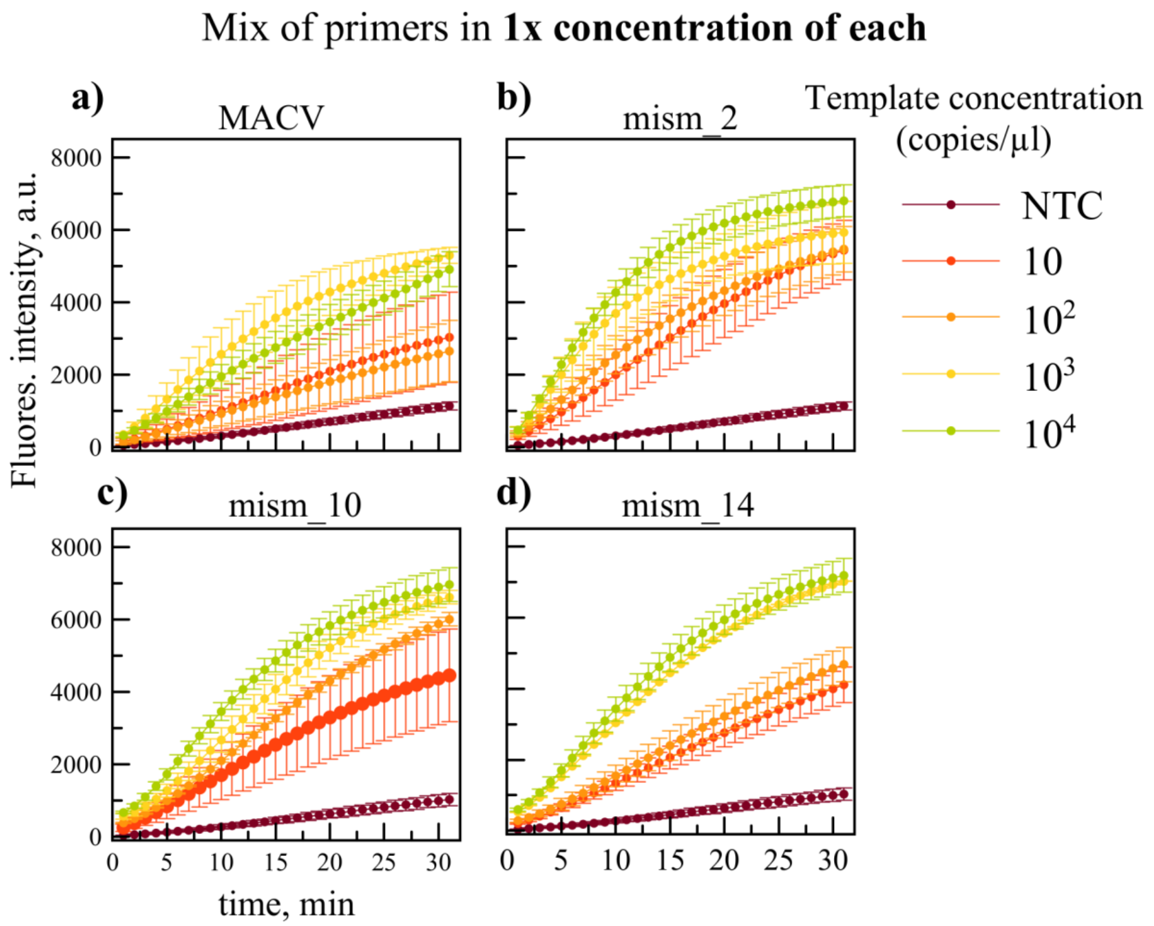

Figure S13. Four-primer RPA/DETECTR assay. Templates MACV, mism_2, mism_10, and mism_14 at different concentrations (10-10^4 copies/µl) are shown. A mix of primers (RPA 1F, RPA 4R, RPA 1F_v2, RPA 4R_v2) was used (each at 1x concentration). The data are presented as the average of three sample replicates with standard deviation.

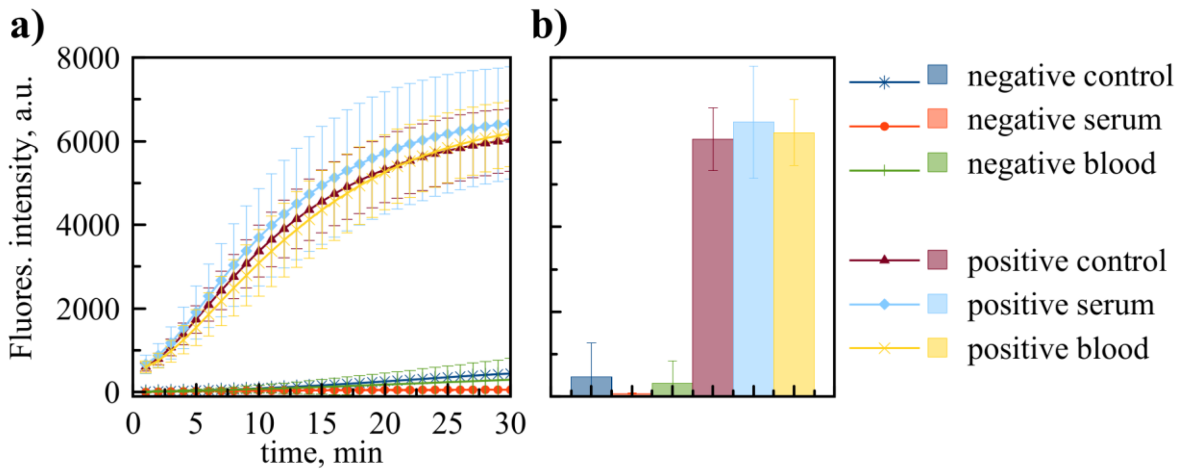

Figure S14. a) RT-RPA/DETECTR assay using primer mix and RNAs extracted from ARP spiked water (control samples), serum, and whole blood samples. b) Comparison of endpoint fluorescent intensity.

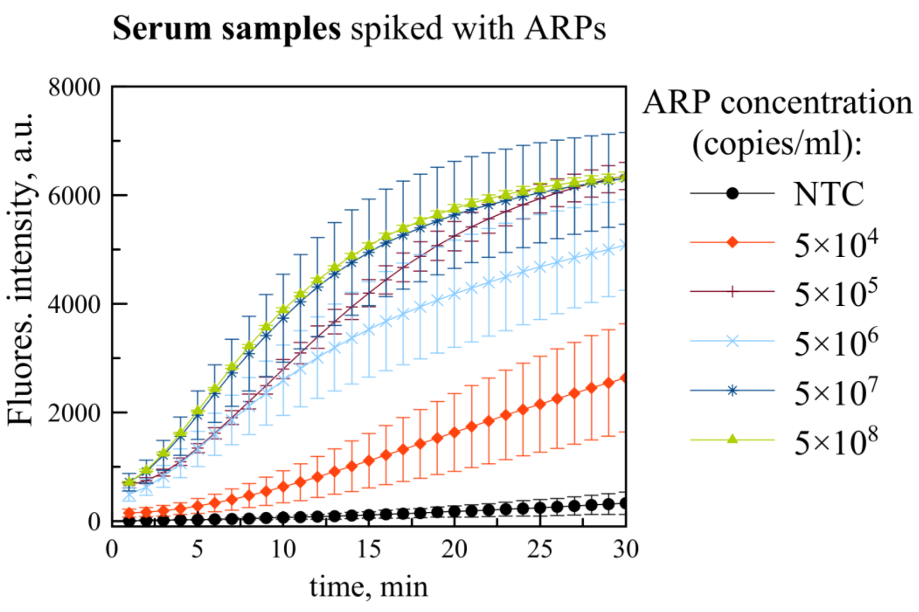

Figure S15. Four-primer RPA/DETECTR assay with RNAs extracted from ARP spiked serum samples at different concentrations.

Table S1. Primer, fluorescent probe, and gRNA sequences used in this work.

Name	Sequence (5`→3`)
MACV_F	TCAACCATCAYGTTGCTTAACG
MACV_R	ATTGACTGAYTTTGCCTTAATGGT
MACV_probe	[R6G] ACCCTCGAAGTTAGAGACAACATACC [BHQ1]
pr_T7_F	TAATACGACTCACTATAGGG
pr_T7_R	GCTAGTTATTGCTCAGCCG
SK_F	AGTTGGAGGACATCAAGCAGCCATGCAAAT
SK_R	TGCTATGTCAGTTCCCCTTGGTTCTCT
SK_probe	[VIC] GAGACCATCAATGAGGAAGCTGCAGAATGG [BHQ-1]
gRNA_1	rUrArArUrUrUrCrUrArCrUrArArGrUrGrUrArGrArUrCrArGrArGrCrUrArUrGrUrUrUrGrUrGrGrArGrGrArUrA
gRNA_2	rUrArArUrUrUrCrUrArCrUrArArGrUrGrUrArGrArUrUrGrGrArGrGrArUrArUrCrGrCrArArUrGrGrGrGrUrArU
ssDNA reporter	[FAM] TTATT [BHQ-1]
RPA 1F	GAGATTCTAACACCCTCGAAGTTAGAGACAAC
RPA 2F	CGTTGCTTAACGAGATTCTAACACCCTCGAAG
RPA 4R	GAATTGACTGACTTTGCCTTAATGGTTAAGAAT
RPA 5R	GACTGACTTTGCCTTAATGGTTAAGAATCAAA
RPA 6R	TGACTTTGCCTTAATGGTTAAGAATCAAAATGT
RPA 1F_v2	GAGATTCTGACACCTTCAAAGTTGGAGACAAC
RPA 4R_v2	GAACTGACTGACTTTGCCTTGATGGTTAAAAGT
MACV_1	CATCAACCATCACGTTGCTTAACG
MACV_2	TGGGGTATGTTGTCTCTAACTTCGAGGGTGTTAGAATCTCGTTAAGCAACGTGAT

	GGTTG
MACV_3	GAAGTTAGAGACAACATACCCCATTGCGATATCCTCCACAAACATAGCTCTGGAAACATT
MACV_4	GAATTGACTGACTTTGCCTTAATGGTTAAGAATCAAAATGTTTCCAGAGCTATGTTTGT
MACV_2_1f	TCAACCATCATGTTGCTTAACGA
MACV_2_2r	GAATTGACTGATTTTGCCTTAATGG
MACV_10_1	CATCAACCACCACGCTGC
MACV_10_2	GGTTATGTTGTCTCCAACTTCGAGGGTGTCAGAATCTCATTAAGCAGCGTGGTGGTTGAT
MACV_10_3	CGAAGTTGGAGACAACATAACCCATTGCGATATCCTCCACAAACATAGCTCTGGTAACAT
MACV_10_4	GAATTAACTGACTTTGCCTTAATGGTTAAAAATCACAATGTTACCAGAGCTATGTTTGTG
MACV_14_1	CATCAACCACCACGTTGC
MACV_14_2	GTTATGTTGTCTCCAACTTTGAAGGTGTCAGAATCTCATTGAGCAACGTGGTGGTTGATG
MACV_14_3	CTTCAAAGTTGGAGACAACATAACCCATTGCAATATCCTCCACAAACATAGCCCTGGAAA
MACV_14_4	GAACTGACTGACTTTGCCTTGATGGTTAAAAGTCAAAATGTTTCCAGGGCTATGTTTGTG

Note: A, G, T, C – DNA nucleobases; Y – cytosine/thymine (pyrimidine) ; rA, rG, rU, rC – RNA nucleobases. Modifications: [R6G] – Rhodamine 6G (9-[2-(Ethoxycarbonyl)phenyl]-N-ethyl-6-(ethylamino)-2,7-dimethyl-3H-xanthen-3-iminium chloride); [FAM] – Fluorescein (3′,6′-dihydroxyspiro[isobenzofuran-1(3H),9′-[9H]xanthen]-3-one); [BHQ-1] – Black Hole Quencher 1 (2-[N-(2-hydroxyethyl)-4-[[2-methoxy-5-methyl-4-[(4-methyl-2-nitrophenyl)diazenyl]phenyl]diazenyl]anilino]ethanol); [VIC] – 5-VIC phosphoramidite.

Table S2. Target sequences used in this work.

Name	Sequence (5`→3`)
MACV	CATCAACCATCACGTTGCTTAACGAGATTCTAACACCCTCGAAGTTAGAGACAACATACCCCATTGCGATATCCTCCACAAACATAGCTCTGGAAACATTTTGATTCTTAACCATTAAGGCAAAGTCAGTCAATTC
mism_2	CATCAACCATCATGTTGCTTAACGAGATTCTAACACCCTCGAAGTTAGAGACAACATACCCCATTGCGATATCCTCCACAAACATAGCTCTGGAAACATTTTGATTCTTAACCATTAAGGCAAAATCAGTCAATTC
mism_10	CATCAACCACCACGCTGCTTAATGAGATTCTGACACCCTCGAAGTTGGAGACAACATAACCCATTGCGATATCCTCCACAAACATAGCTCTGGTAACATTGTGATTTTTAACCATTAAGGCAAAGTCAGTTAATTC
mism_14	CATCAACCACCACGTTGCTCAATGAGATTCTGACACCTTCAAAGTTGGAGACAACATAACCCATTGCAATATCCTCCACAAACATAGCCCTGGAAACATTTTGACTTTTAACCATCAAGGCAAAGTCAGTCAGTTC
GTOV	AAAAGCTTGAAGCCCTCTGTTAAGACCATCGGTATTTCAACTTGTGAAAGCAATGCCTC

	CTTCTGATAAGACAGTTCAGGTATATCAGGTACTTGCCTTCTGATGAAGTCCCTCAAAACTACAACTTTTTCATCCATTGTCAAATTTCGCCTAGGAT
SABV	ATTGAAAGCCACTTTTCATTGGTGGTCAGTTTTTGCCTGATCAACTTATTAGCAGTGAGGATGTCCAACACCCGATCATTGACAAGTAATATCATTTGTCCAACTATTAATCCTGCATTCTTTAGGTTCCTGATAA
JUNV	TGGGCTGATAAACTTGTTTAATTTAGATGATAAAAATTCATGGAAGCACACCATTTCCAGCAGTTCTGTTCTGTCTTGACACTTTTCATCACTAAGGCAAGGAATCTTTATAAGGCTAACCTGGTCATCACTGGAGGTATAAGTGACAGGTATCACATCA
HENV	AGGAAAGTGAGACCAGAAGATGGGCAAAGTATGTTCAACAAAAGAGGGTCAATCCATTCTTTGCCTTAACCCAGCAATGGCTGACAGAGATGAGGAATCTCCTCTCACAAAGTCTCTCAGTCAGAAAATTCATGGTGGAAATTCTGATGGAGGTAAAGAA
NPV	CCCAAAGTATCACTGATTGACACATCCAGTACCATTACTATCCCAGCTAACATTGGGCTGTTAGGTTCAAAGATCAGCCAGTCGACTGCAAGTATAAATGAGAATGTGAATGAAAAATGCAAATTCACACTGCCTCCCTTGAAAATCCACGAATGTAACATTTCTTGTC

Table S3. PCR reaction mix and conditions to obtain a linear DNA positive control.

Component	*Volume per reaction, µl*
Taq M master mix (Alkor Bio, Russia)	25
10 µM forward/reverse primers (pr_T7_F/pr_T7_R)	2.5/2.5
ultrapure water	19
plasmid	1
Protocol: 95°C 15 min; 27 cycles (95°C 30 sec, 56°C 30 sec, 72°C 2.5 min); 72°C 5 min	

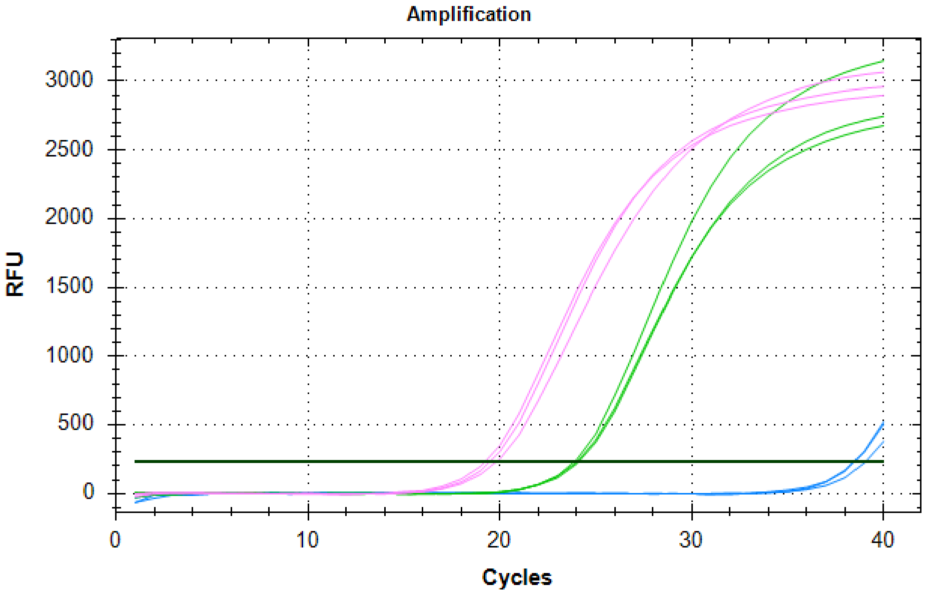

Figure S16. Selection of armored RNA particle (ARP) fraction by real-time RT-PCR: first (blue), second (green), third (purple).

Table S4. Reaction mix and conditions for ddPCR using the One-Step RT-ddPCR Advanced Kit for Probes.

Component	*Volume per reaction, μl*
Supermix	5
reverse transcriptase	2
300 mM DDT	1
10 μM forward/reverse primers (SK_F/SK_R)	1.8/1.8
10 μM fluorescent probe (SK_probe)	0.56
ultrapure water	2.84
extracted RNA	5
Protocol: 50°C 60 min; 95°C 10 min; 40 cycles (95°C 30 sec, 55°C 1 min); 98°C 10 min	

Table S5. Reaction mix for the DETECTR assay.

Component	*Volume per reaction, μl*
1 μM Lba Cas12a (New England Biolabs, USA)	2.5
1 μM gRNA	2.5
10x NEBuffer™ r2.1 (New England Biolabs, USA)	2.5
10 μM ssDNA reporter	2.5
ultrapure water	14
DNA template	1

Table S6. Reaction mix for the RPA assay using TwistAmp® Liquid Basic kit reagents (TwistDx™, UK).

Component	*Volume per reaction, μl*
10 μM forward/reverse primers	0.72/0.72
2x Reaction Buffer	7.5
dNTPs, 10 mM of each (New England Biolabs, USA)	0.675
10x Basic E-mix	1.5
20x Core Reaction Mix	0.75
ultrapure water	1.385
280 мM magnesium acetate	0.75
DNA template	1

Table S7. Reaction mixes for the combined single-tube RT-RPA/DETECTR assay.

Component	*Volume per reaction, μl*
RT-RPA mix (total volume 14 μl)	
10 μM forward/reverse primers	0.72/0.72
2x Reaction Buffer	7.5
dNTPs, 10 mM of each (New England Biolabs, USA)	0.675

10x Basic E-mix	1.5
20x Core Reaction Mix	0.75
ultrapure water	1.085
280 мM magnesium acetate	0.75
200,000 U/ml M-MuLV (New England Biolabs, USA)	0.3
DETECTR mix (total volume 10 µl)	
1 µM Lba Cas12a (New England Biolabs, USA)	2.5
1 µM gRNA	2.5
10x NEBuffer™ r2.1 (New England Biolabs, USA)	2.5
10 µM ssDNA reporter	2.5
RNA	1
